# Discovery of record-breaking high-*T*_c_ superconductivity among 5*d* transition-metal-oxides up to 17.8 K in ReO_3_

**DOI:** 10.1093/nsr/nwag425

**Published:** 2026-07-10

**Authors:** Pengfei Shan, Tenglong Lu, Ziyi Liu, Yuanyuan Jiao, Jiajia Feng, Pengtao Yang, Liang Ma, Yoshiya Uwatoko, Xiaoli Dong, Bosen Wang, Bin Chen, Miao Liu, Jianping Sun, Jinguang Cheng

**Affiliations:** Beijing National Laboratory for Condensed Matter Physics and Institute of Physics, Chinese Academy of Sciences, Beijing 100190, China; School of Physical Sciences, University of Chinese Academy of Sciences, Beijing 100190, China; Beijing National Laboratory for Condensed Matter Physics and Institute of Physics, Chinese Academy of Sciences, Beijing 100190, China; School of Physical Sciences, University of Chinese Academy of Sciences, Beijing 100190, China; Beijing National Laboratory for Condensed Matter Physics and Institute of Physics, Chinese Academy of Sciences, Beijing 100190, China; School of Physical Sciences, University of Chinese Academy of Sciences, Beijing 100190, China; Beijing National Laboratory for Condensed Matter Physics and Institute of Physics, Chinese Academy of Sciences, Beijing 100190, China; Faculty of Science, Wuhan University of Science and Technology, Wuhan 430065, China; Center for High Pressure Science and Technology Advanced Research, Beijing 100193, China; Beijing National Laboratory for Condensed Matter Physics and Institute of Physics, Chinese Academy of Sciences, Beijing 100190, China; School of Physical Sciences, University of Chinese Academy of Sciences, Beijing 100190, China; Beijing National Laboratory for Condensed Matter Physics and Institute of Physics, Chinese Academy of Sciences, Beijing 100190, China; Key Laboratory of Materials Physics, Ministry of Education, School of Physics and Microelectronics, Zhengzhou University, Zhengzhou 450052, China; Department of Advanced Materials Science, Graduate School of Frontier Sciences, University of Tokyo, Chiba 277-8581, Japan; Department of Natural Sciences, Faculty of Science and Engineering, Tokyo City University, Tokyo 158-8557, Japan; Beijing National Laboratory for Condensed Matter Physics and Institute of Physics, Chinese Academy of Sciences, Beijing 100190, China; School of Physical Sciences, University of Chinese Academy of Sciences, Beijing 100190, China; Beijing National Laboratory for Condensed Matter Physics and Institute of Physics, Chinese Academy of Sciences, Beijing 100190, China; School of Physical Sciences, University of Chinese Academy of Sciences, Beijing 100190, China; Center for High Pressure Science and Technology Advanced Research, Beijing 100193, China; Beijing National Laboratory for Condensed Matter Physics and Institute of Physics, Chinese Academy of Sciences, Beijing 100190, China; School of Physical Sciences, University of Chinese Academy of Sciences, Beijing 100190, China; Beijing National Laboratory for Condensed Matter Physics and Institute of Physics, Chinese Academy of Sciences, Beijing 100190, China; School of Physical Sciences, University of Chinese Academy of Sciences, Beijing 100190, China; Beijing National Laboratory for Condensed Matter Physics and Institute of Physics, Chinese Academy of Sciences, Beijing 100190, China; School of Physical Sciences, University of Chinese Academy of Sciences, Beijing 100190, China

**Keywords:** 5*d* transition-metal oxide, high-pressure physics, light-element superconductivity, first-principles calculations, electron-phonon coupling

## Abstract

As an *A*-site-vacant perovskite-type oxide, ReO_3_ undergoes sequential pressure-driven structural phase transitions associated with rotations of ReO_6_ octahedra. In this work, we report the discovery of a superconducting *T*_c_(*P*) dome for the rhombohedral-I phase of ReO_3_ in the pressure range 12–39 GPa. The rhombohedral-I phase is featured by the nearly close-packed oxygen layers intercalated with Re cations, which is reminiscent of superhydride superconductors. The observed maximum *T*_c_ (∼ 17.8 K) sets the record of high *T*_c_ among 5*d* transition-metal-oxides. First-principles calculations reveal that the strong hybridizations between Re-5*d* and O-2*p* orbitals significantly enhance the density of states at the Fermi level and the close-packed oxygen framework directly enhances the electron-phonon coupling. We attributed the observed record high-*T*_c_ to the synergistic effect of strong hybridizations and oxygen sublattice contribution. The present work establishes a rare case that the light-element oxygen lattice plays a crucial role in assisting the emergence of high-*T*_c_ superconductivity and provides more clues for further exploring new superconductors in 5*d* transition-metal oxides via high-pressure or heterostructure engineering.

## Teaser

The record-high superconductivity among 5d transition-metal oxides, discovered in pressurized ReO3, is shown to be driven by a synergistic interplay between strong Re-O orbital hybridization and the close-packed light-element oxygen lattice.

## INTRODUCTION

Since the discovery of superhydride superconductors with *T*_c_ up to 260 K [1,2], much effort has been devoted to elucidate its origin and the results indicate that hydrogen plays a crucial role within the framework of electron-phonon coupling (EPC) mechanism [3], such as H_3_S [4]. Similar features have also been identified in other light-element superconductors such as MgB_2_ [5,6] and NaC_8_ [7], which exhibit layered-honeycomb-boron and layered-carbon frameworks, and show high *T*_c_ reaching ∼ 39 K at ambient pressure (AP) and 28 K at 14 GPa, respectively. In addition, the discovery of mixed-valence main-group perovskite oxides bismuthates [8–10] and antimonates [11] superconductors have also received lots of attention due to suppression of competing charge orders and the important role of oxygen lattice in driving superconductivity. Experimental investigations and theoretical calculations on these systems have identified strong metal-oxygen covalency and enhanced EPC as the possible origin of high *T*_c_ [12,13]. Recently, superconductivity has been discovered in simple binary oxides such as rock-salt LaO, which shows *T*_c_ ∼ 5–6 K at AP and was further enhanced to 12.7 K under pressure. However, the density of states (DOS) at the Fermi level [*N*(*E*_F_)] mainly comes from La-5*d* electrons [14]. Realizing unconventional bonding features in metallic oxides remains challenging due to the strong electronegativity of oxygen, yet it continuously inspired researchers to discover favorable oxide superconducting systems.

**Figure 1.**
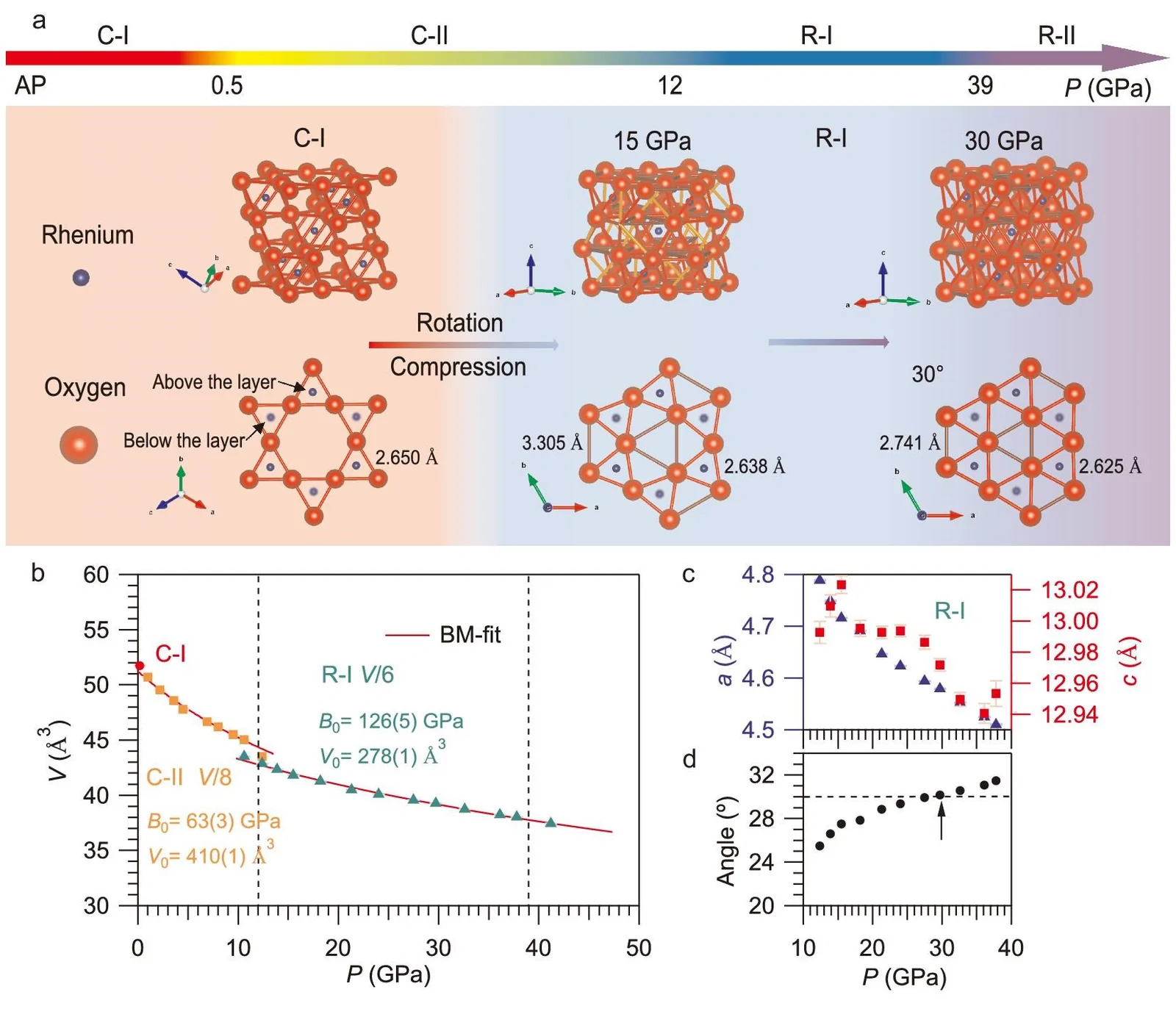
Crystal structure evolution and lattice parameters of ReO_3_ under high pressure. (a) The horizontal arrow on the top shows the pressure-induced structure phase transitions of ReO_3_ from C-I to R-II phases at 0.5 GPa, 12 GPa, and 39 GPa. The bottom part displays the coupled rotation of oxygen layers from Kagome lattice to triangular lattice in the (111) plane. Bond lengths were obtained from DFT calculations, as shown in Table S2. The solid lines connecting the oxygen atoms serve as visual guides to illustrate the structural evolution and densification of the oxygen sublattice. They represent the nearest-neighbor O-O distances. (b) Pressure dependence of volume for different phases of ReO_3_. Solid lines represent the fitting by using Birch-Murnaghan (BM) equation of state. (c) Pressure dependence of the lattice parameters *a* and *c* of ReO_3_. (d) Pressure dependence of the coupled rotation angle of ReO_6_ octahedra in the R-I phase. The arrow indicates the critical pressure where the oxygen layers form the perfect hexagonal-close-packed lattice.

For the exploration of oxide analogues of hydride superconductors, we turn to 5*d* transition-metal-oxide ReO_3_, a high oxygen molar ratio and purple-colored metallic oxide with high conductivity comparable to that of copper and silver [15]. It adopts the A-site-vacant perovskite structure with corner-shared ReO_6_ octahedra [16]. Spectroscopy measurements confirm that the excellent conductivity originates from the bond covalency between Re-*t*_2g_ and O*-p*_π_ orbitals through the linear Re-O-Re bonds [15,17] and the hybridizations further raise the 2*p* orbitals of oxygen to the Fermi level. On the other hand, ReO_3_ was predicted to be a superconductor at AP [18], but no superconductivity was observed down to 20 mK. Intriguingly, ReO_3_ undergoes a sequential pressure-driven structure phase transitions from cubic-I ($Pm\!\!\! \bar{\,\,\,3}m$) (C-I) to cubic-II ($Im\!\!\! \bar{\,\,\,3}$) (C-II) at ∼ 0.5 GPa, to monoclinic (*C*2/*c*) at ∼ 3 GPa, to rhombohedral-I ($R\!\!\! \bar{\,\,\,3}c$) (R-I) at ∼ 12 GPa, and to rhombohedral-II ($R\!\!\! \bar{\,\,\,3}c$) (R-II) at ∼ 39 GPa [18–23]. But no consensus has been reached on the accurate high-pressure phases and critical transition pressures, as summarized in Table S1 of the Supplemental Information (SI). Nevertheless, the presence of the R-I phase between 12 and 39 GPa has been well established, as shown in Fig. 1.

Inspired by realizing strong covalency between transition-metal and oxygen that would facilitate a higher *T*_c_, we investigated the physical properties of 5*d* transition-metal-oxide ReO_3_. By combining high-pressure structure characterizations and transport measurements, we created a high-*T*_c_ record with 17.8 K in the R-I phase of ReO_3_ at 35 GPa among 5*d* transition-metal-oxides. The theoretical analyses further demonstrate that the superconductivity originates from a synergistic effect of pressure-enhanced Re-O hybridization and the substantial contributions to the EPC from close-packed oxygen stacking lattice. The R-I phase of ReO_3_ constitutes a promising oxide analogue of hydride superconductors. Thus, our work would stimulate more effort to further explore new high-*T*_c_ superconductors among 5*d* transition-metal-oxides via high-pressure or heterostructure engineering.

## RESULTS

### High-pressure structural evolution of ReO_3_

Figure 1 shows the pressure-induced evolution of the crystal structure of ReO_3_, which is particularly intriguing, especially when viewed from the perspective of the oxygen sublattice. The R-I phase emerges from the C-I phase through coupled rotations of ReO_6_ octahedra along the ⟨111⟩ direction [20]. As illustrated in the bottom panel of Fig. 1a, the oxygen atoms progressively evolve from a perfect kagome lattice in the C-I phase into a distorted triangular lattice in the R-I phase. With further increasing pressure, this lattice becomes more uniform and eventually attains a hexagonal close-packed configuration at a rotation angle of 30° above 30 GPa. Under higher pressures, volume reduction can no longer be accommodated solely by octahedral rotations, leading to an additional structural transition into the R-II phase above 39 GPa [20,21]. Notably, such close-packed oxygen layers in the R-I phase are rarely observed in other oxide materials.

These sequential structural phase transitions were confirmed by our high-pressure synchrotron X-ray diffraction (SXRD) measurements on ReO_3_. As displayed in Fig. S1a, the SXRD patterns can be indexed by using the C-I, C-II, mixed C-II/R-I, and R-I phase in different pressure ranges up to 30 GPa, respectively. Moreover, some new diffraction peaks emerge at 36.1 GPa, Fig. S1b, which indicates the occurrence of structure phase transition to R-II phase. We refined the structure parameters of single-phase regions for C-II and R-I, and the obtained pressure-volume (*P*-*V*) relations were displayed in Fig. 1b. As shown in Fig. 1c, the coupled rotations of ReO_6_ octahedra exhibit a significant anisotropic compression, i.e. the *a*-axis shrinks by more than 6% while *c*-axis is only reduced by 0.6% at 40 GPa. By assuming rigid rotations of the octahedra, the rotation angle can be estimated by the ratio of *a/c*. We can see that the rotation angle gradually increases from 25° to 30° in the R-I phase, Fig. 1d. With further increasing pressure, the octahedra rotates inward by nearly 2°, which destabilizes the R-I phase at ∼ 39 GPa and leads to a new structure phase transition to R-II phase. The above results provide a quantitative description of octahedral rotations by involving the structural phase transitions associated with the R-I phase.

### Experimental characterization of superconductivity in ReO_3_

**Figure 2.**
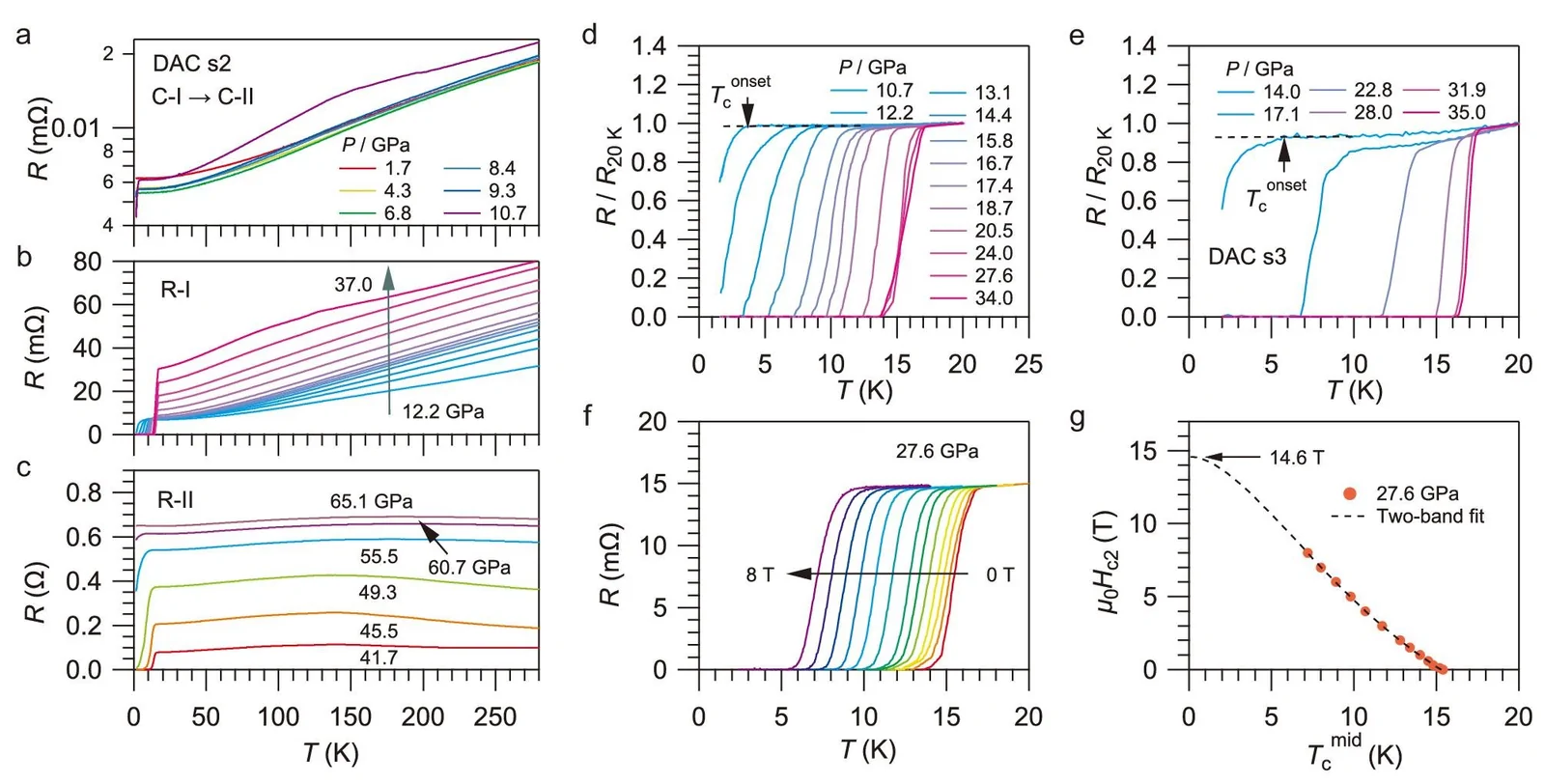
Electrical transport properties measured under high pressure. Temperature dependence of resistance of ReO_3_ (DAC s2) measured at various pressures: (a) from 1.7 to 10.7 GPa, (b) from 12.0 to 37.0 GPa, and (c) from 41.7 GPa to 65.1 GPa. (d and e) Normalized resistance *R*/*R*_20 K_ of ReO_3_ (DAC s2 and s3) under various pressures up to 35 GPa. (f) Temperature dependence of resistance (DAC s2) under various magnetic fields at 27.6 GPa. (g) Temperature dependence of the upper critical field *μ*_0_*H*_c2_(*T*) at 27.6 GPa (DAC s2). The dashed line represents the fitting results by using WHH two-band model.

Then we performed high-pressure transport measurements on ReO_3_ single crystals by employing cubic anvil cell (CAC) and diamond anvil cell (DAC). The samples were labeled as s1 for CAC and s2 to s3 for DAC, and more details can be found in Table S3. As shown in Fig. S2a and b, the resistance and Hall resistance measured at AP are consistent with previous reports [24]. As we can see in Figs 2a–c, S2c and S3a–c, the normal-state resistance increases gradually both in C-II and R-I phases under high pressures. Surprisingly, an obvious drop gradually emerges in resistance below 2 K above 12 GPa and the zero resistance can be observed at 13 GPa, which indicates the emergence of superconductivity in the R-I phase, inset of Fig. S2c. More detailed evolution of superconducting transition can be clearly tracked from normalized resistance *R*(*T*)/*R*(20 K) in Fig. 2d and e. Here, we define the temperature of deviation from normal-state resistance as *T*_c_^onset^ and the zero resistance as *T*_c_^zero^. With increasing pressure gradually, the superconducting *T*_c_ exhibits continuous enhancement for both s2 and s3. Moreover, the maximum *T*_c_^onset^ and *T*_c_^zero^ can reach about 17.8 K and 16.3 K at 35 GPa. To the best of our knowledge, the observed maximum *T*_c_^onset^ and *T*_c_^zero^ of ReO_3_ produces the record of the highest *T*_c_ among 5*d* transition-metal oxides.

To further characterize superconducting properties, we measured *R*(*T*) under various magnetic fields at each pressure. As displayed in Figs 2f and S4, the superconducting transition gradually shifts to lower temperatures with increasing magnetic field. Here, we define *T*_c_ as the temperature where the resistance drops to half of normal state resistance *R_n_*. In Figs 2g and S5, *μ*_0_*H*_c2_(*T*) exhibits obvious upward curvature at low fields, which indicates multi-band feature of ReO_3_. The *μ*_0_*H*_c2_(*T*) can be well fitted by the Werthamer-Helfand-Hohenberg (WHH) two-band model [25], and the obtained *μ*_0_*H*_c2_(0) ∼ 14.6 T at 27.6 GPa is smaller than Pauli limit *μ*_0_*H*_p_^BCS^ = 1.84*T*_c_ = 28.3 T, Fig. 2g. The analysis of upper critical field indicates that the emergent superconductivity in ReO_3_ has multiband features that may originate from the hybridization between Re-5*d* and O-2*p* orbitals.

In addition, we performed magnetic susceptibility measurements to confirm the bulk nature of pressure-induced superconductivity in ReO_3_. As shown in Fig. S6, systematic pressure-dependent ZFC/FC and field-dependent *M*-*T* measurements confirm the bulk superconductivity. The superconducting volume fraction exceeds 78% at 23 GPa for s4 and approaches or even slightly exceeds 100% at 25 and 30 GPa for s5 after properly corrected for demagnetization effects. The detailed calculations can be seen in the Methods section.

### Phase diagram of superconductivity in ReO_3_

**Figure 3.**
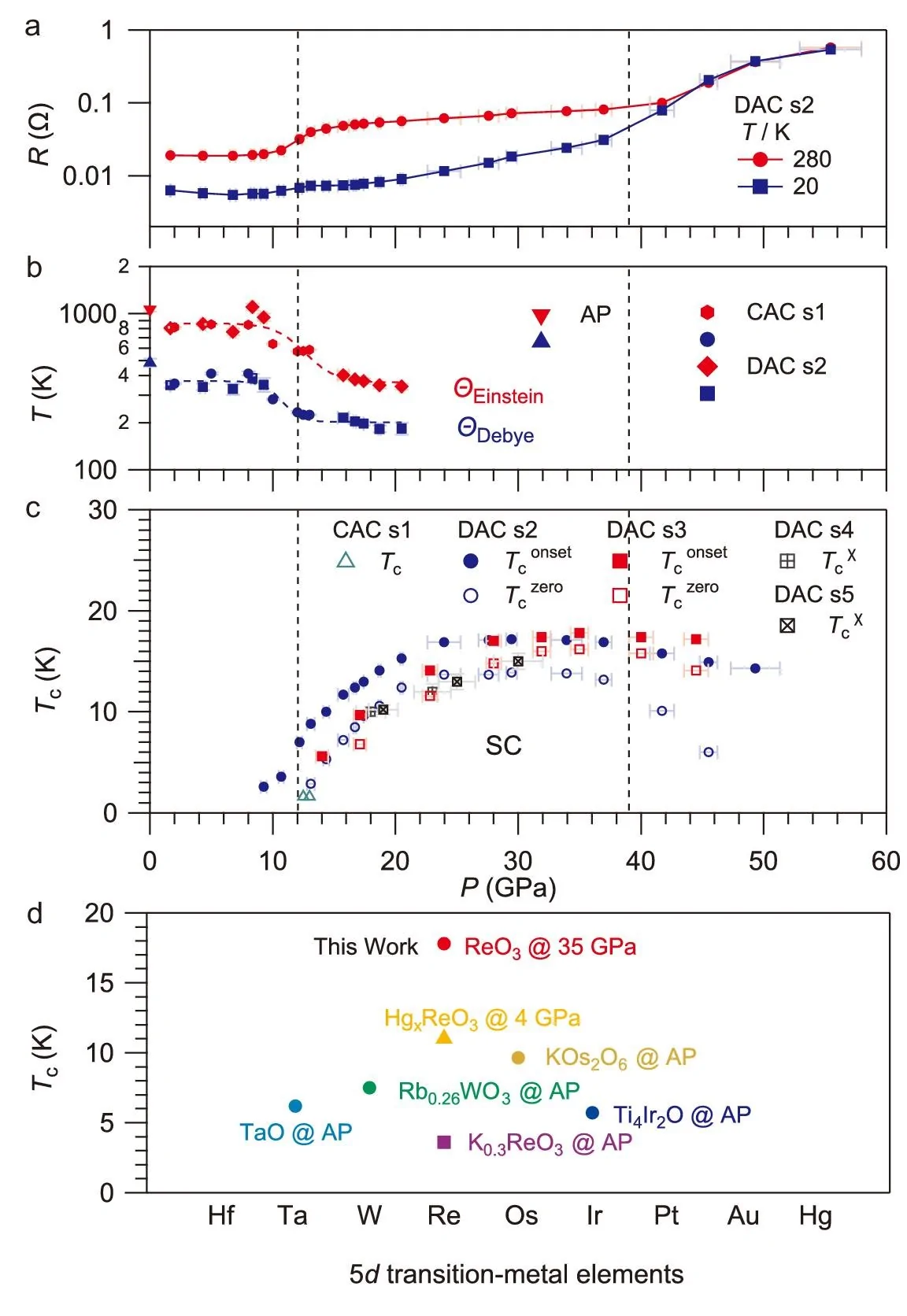
Electrical and superconducting properties of ReO_3_. Pressure dependences of (a) the resistance of ReO_3_ at 20 and 280 K, (b) the calculated Debye and Einstein temperatures from fitting to resistance by using modified Bloch-Grüneisen equation, (c) superconducting transition temperatures of ReO_3_ in different runs. The dashed line marks the phase boundaries. (d) Typical 5*d* transition metal oxide superconductors and their maximum superconducting transition temperatures, such as TaO (*T*_c_ = 6.2 K @ AP) [27], Rb_0.26_WO_3_ (*T*_c_ = 7.5 K @ AP) [28], K_0.3_ReO_3_ (*T*_c_ = 3.6 K @ AP) [29], Hg_*x*_ReO_3_ (*T*_c_ = 11.1 K @ 4 GPa) [30], ReO_3_ (*T*_c_ = 17.8 K @ 35 GPa) (our work), KOs_2_O_6_ (*T*_c_ = 9.65 K @ AP) [31], and Ti_4_Ir_2_O (*T*_c_ = 5.7 K @ AP) [32].

To further elucidate the correlation between structural phase transitions and transport properties, we extract the resistance values at 280 K and 20 K and lattice dynamics information by analyzing resistance under high pressures. As displayed in Fig. 3a, the evolution of resistance at 280 K and 20 K clearly illustrates the phase boundary of successive structure phase transitions. In addition, we can clearly see that the *R*(*T*) curves at both ambient and high pressures can be well fitted by modified Bloch-Grüneisen equation [18,26] with the fitting results shown in Fig. 3b. As can be seen, both Debye and Einstein temperatures, *Θ*_D_ and *Θ*_E_, exhibit a dramatic drop above ∼ 10 GPa where the structure phase transition to R-I phase takes place. This feature is correlated with a phonon softening effect and is expected to assist the emergence of superconductivity.

Based on the obtained *T*_c_^onset^, *T*_c_^zero^ and *T*_c_*^χ^* of ReO_3_ under various high pressure, the temperature-pressure phase diagram can be constructed in Fig. 3c. The superconducting *T*_c_(*P*) is featured by a broad dome with maximum *T*_c_ ≈ 17.8 K at 35 GPa. Combined with structural characterizations, we can see that the emergence of superconducting phase is accompanied by a structure phase transition and the maximum *T*_c_ is achieved when the oxygen layers form a perfect hexagonal close packing. Figure 3d summarizes the reported *T*_c_ of 5*d* transition-metal oxide superconductors at AP and high pressure [27–32], which highlights the highest *T*_c_ ∼ 17.8 K of ReO_3_. The present work thus will inspire researchers to explore high-*T*_c_ superconductivity in 5*d* transition-metal-oxides by using high-pressure and heterostructure engineering.

### Theoretical insights on superconductivity of ReO_3_

**Figure 4.**
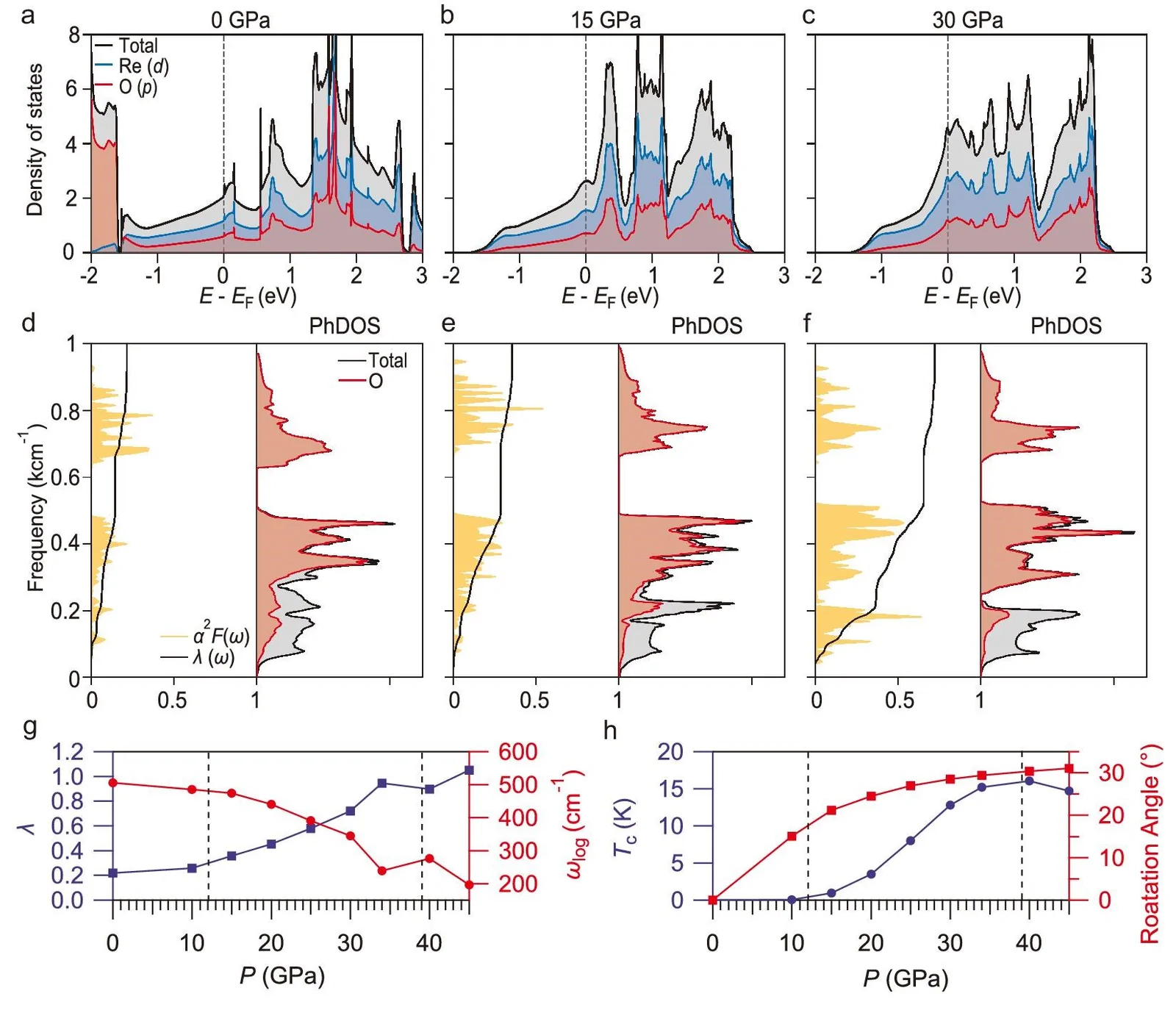
Electronic density of states, electron-phonon coupling and superconducting properties of ReO_3_ under high pressures. (a–c) Total density of states (DOS) and partial DOS for ReO_3_ at 0, 15, and 30 GPa. Energy zero represents the Fermi level. (d–f) Eliashberg spectral function *α^2^F*(*ω*), and cumulative frequency-dependent EPC constant λ(*ω*), total phononic density of states (PhDOS) and partial PhDOS at 0, 15, and 30 GPa. (g) Calculated λ and ${\omega }_{{\mathrm{log}}}$ as a function of pressure. (h) *T*_c_ and rotation angle as a function of pressure. The dashed line marks the phase boundaries.

To further explore the mechanism of pressure-induced high-*T*_c_ superconductivity in ReO_3_, we performed theoretical calculations at selected pressures. First, the crystal structure was fully relaxed from 0 to 45 GPa to mimic the structural evolutions of ReO_3_. The DFT calculations further demonstrate that the rotations of ReO_6_ octahedra in the C-I phase along the <111> axis gradually densifies oxygen layers and then transforms to R-I phase above 10 GPa, which is consistent with experimental results. An ideal hexagonal close packing is realized with rotation angle of 30° around 30 GPa. Figure 4a–c shows the electronic DOS at 0 GPa (cubic-I), 15 and 30 GPa (R-I) with different rotation angles. With increasing pressure gradually, the *N*(*E*_F_) shows an increase at 15 GPa and is almost doubled at 30 GPa. Moreover, the Re-5*d* and O-2*p* orbitals are strongly hybridized, and O-2*p* orbitals have considerable contributions to the *N*(*E*_F_) at 15 and 30 GPa in the R-I phase. Moreover, the Crystal Orbital Hamilton Population (COHP) analyses reveal the above evolution that the Re-O interactions exhibit a distinct antibonding character near the Fermi level and show continuous enhancement with pressure, Fig. S7. As displayed in Fig. S8, the integrated COHP (-ICOHP) displays significant increase. It explicitly demonstrates that the evolution of Re-O bonding directly driven the enhancement of *N*(*E*_F_). Figure 4d–f displays the calculated phonon DOS, Eliashberg spectral function *α*^2^*F*(*ω*) and cumulative frequency-dependent EPC constant λ(*ω*) at 0, 15 and 30 GPa. It can be clearly seen that both Re and O have large contributions to the lower frequency phonons (<250 cm^−1^), while the higher frequency phonons (>250 cm^−1^) were dominated by oxygen. However, the *α*^2^*F*(*ω*) exhibits a pronounced spectra weight shift to lower-frequency region, which indicates the clear phonon softening of Re-O bonding branches upon entering the R-I phase. Based on the Eliashberg theory, the phonon softening is consistent with a sudden drop of Debye and Einstein temperatures observed in experiments and can dramatically enhance the EPC constant. As shown in Fig. 4g and h, the λ exhibits a fourfold enhancement from ∼ 0.2 at 0 GPa to ∼ 0.9 at 34 GPa, and the *T*_c_ emerges in the R-I phase and displays a dome shape with the maximum reaching ∼ 15 K at 34 GPa when the rotation angle approaches 30°, which is consistent with the experimental results.

## DISCUSSION

The main result of present study is the observation of superconductivity with maximum *T*_c_ ∼ 17.8 K in the high-pressure R-1 phase of ReO_3_ at 35 GPa, which sets the highest *T*_c_ record among the reported 5*d* transition-metal oxide superconductors. As we know, the 3*d* transition-metal oxides have strong electron correlations and give birth to the cuprates [33,34] and nickelates [35,36] high-*T*_c_ families. In contrast, 5*d* transition-metal elements have more spatially extended orbitals, strong spin-orbital couplings and weak electron correlations, and often exhibit low *T*_c_ superconductivity in its oxides. ReO_3_ stands out with strong hybridization between Re-5*d* and O-2*p* states and exhibits a maximum *T*_c_ of ∼ 17.8 K. Our work indicates that the rotations of ReO_6_ octahedra under pressure strongly enhances the hybridizations between Re-5*d* and O-2*p* orbitals. The structural evolution gradually pushes the Re-O antibonding states to the Fermi level and simultaneously increases the *N*(*E*_F_). Thus, the emergence of high-*T*_c_ superconductivity in R-I phase of ReO_3_ represents a compelling example of phonon-mediated superconductivity driven by a synergistic effect between the heavy 5*d* element and the light oxygen sublattice.

To further quantify the role of light element in boosting high-*T*_c_ superconductivity, we make a comparison between ReO_3_ and representative covalent hydride superconductor H_3_S. For H_3_S, the high-*T*_c_ superconductivity is not merely driven by hydrogen; sulfur also plays a critical role for constructing strong covalent network and enhances the EPC [37]. While in ReO_3_, the extended Re-5*d* orbitals provide necessary electronic framework, and the oxygen contributes ∼ 27% to *N*(*E*_F_) (∼ 42% for H in H_3_S) and ∼ 50% to λ at 30 GPa (∼ 67% for H in H_3_S) [38]. Therefore, ReO_3_ can be regarded as an oxide analogue of hydride superconductors in R-I phase, which share high similarity of pressure-optimized covalent hybridizations. However, the lattice dynamics of oxygen were intrinsically restricted the phonon frequencies compared to the lighter hydrogen and further set the upper limit for the maximum *T*_c_.

Inspired by the observation of high-*T*_c_ superconductivity in R-I phase of ReO_3_, our work highlights the promising routes to explore new superconductors in 5*d* transition-metal-oxides via high-pressure or heterostructure engineering. It should be noted that the contribution of oxygen (∼ 50%) to the EPC is currently derived from DFT calculations and the direct experimental verifications would be provided with the oxygen isotope effect measurements, i.e. comparing the *T*_c_ of Re^16^O_3_ and Re^18^O_3_ under high pressures.

## CONCLUSION

In summary, we revealed a dome-shaped superconducting *T*_c_(*P*) in the R-I phase of ReO_3_ over a wide pressure range of 12–39 GPa, and the maximum *T*_c_ ≈ 17.8 K sets a record of high-*T*_c_ among 5*d* transition-metal oxides. The enhancement of *N*(*E*_F_) originating from enhanced Re-O hybridizations and the phonon modes associated with close-packed oxygen sublattice are essential to the emergence of superconductivity. The present study demonstrates that the synergistic effect between extended 5*d* orbitals of Re and the light element O mediates superconductivity in ReO_3_. The present work provides important clues for exploring new high-*T*_c_ superconductors in 5*d* transition-metal-oxides via high-pressure study or heterostructure engineering and also offers a new route for designing high-*T*_c_ superconductors in oxygen-rich systems.

## METHODS

### Electrical transport measurements

High-quality ReO_3_ single crystals were synthesized via the chemical vapor transport method [39]. To comprehensively evaluate the intrinsic superconductivity and its sensitivity to pressure homogeneity, the high-pressure transport measurements were systematically conducted using different apparatuses and pressure-transmitting media (PTM). Measurements in the lower pressure range were performed by using palm-type CAC with liquid glycerol as the PTM (s1). The multi-anvil triaxial compression of the CAC provides a highly isotropic and nearly ideal hydrostatic environment, allowing the emergence of superconductivity to coincide precisely with the structural transition pressure. Higher pressure measurements were carried out by using BeCu-type DACs. To compare hydrostatic effects, two different PTM were employed in the DAC experiments: soft solid KBr (s2) and liquid Daphne 7373 (s3). While Daphne 7373 offers relatively good hydrostaticity under uniaxial compression, solid KBr provides relatively poor hydrostatic conditions, generating significant internal pressure gradients and local shear stresses. For sample s2, local regions with higher pressures show the superconducting transition at lower pressures. Pressure values inside the sample chamber were initially determined at room temperature by the ruby fluorescence method [40] or from the first-order diamond Raman peak shift [41]. The pressure variations upon cooling down in both DAC and CAC have been characterized by comparing the pressure calibration curves at room temperature and low temperatures.

### X-ray diffraction measurements

The high-pressure SXRD experiments were performed at the BL15U1 station of the Shanghai Synchrotron Radiation Facility (SSRF) (λ *=* 0.6199 Å). Here, the ethyl-alcohol mixture with a ratio of 4:1 was used as a PTM. Using the geometric parameters calibrated by a CeO_2_ standard, the DIOPTAS software package [42] was used to integrate the powder diffraction patterns and then convert them to one-dimensional profiles. Rietveld refinements of XRD patterns were conducted using GSAS with EXPGUI packages [43]. The *P*-*V* relationship is fitted by using the Birch-Murnaghan equation:


(1) \begin{eqnarray*}
{{P}}\left( {{V}} \right) &=& \frac{{3{{{B}}}_0}}{2}\left[ {{{\left( {\frac{{{{{V}}}_0}}{{{V}}}} \right)}}^{7/3} - {{\left( {\frac{{{{{V}}}_0}}{{{V}}}} \right)}}^{5/3}} \right]\\
&&\times \left\{ {1 + \frac{3}{4}\left( {{{B}}_0^{\prime} - 4} \right)\left[ {{{\left( {\frac{{{{{V}}}_0}}{{{V}}}} \right)}}^{2/3} - 1} \right]} \right\},\\
\end{eqnarray*}

where *V*_0_ is the cell volume at AP, *V* is the volume under pressure *P, B*_0_ is the bulk elastic modulus, and ${{B}}_0^{\prime} = 4$ is the derivative of the bulk elastic modulus at zero pressure. If we assume the rotation is rigid, the rotation angle ${{\theta }}$ can be estimated by the change of *a*/*c* via the following relationship:


(2) \begin{eqnarray*}
{{\theta }} = \arccos \left( {\sqrt 6 {{a}}/{{c}}} \right).\
\end{eqnarray*}

### WHH two-band model

The Werthamer-Helfand-Hohenberg (WHH) two-band model in the dirty limit [25]:


(3) \begin{eqnarray*}
&&{{{a}}}_0[ {\ln {{t}} + {{U}}( {{{{D}}}_1{{h}}} )} ][ {\ln {{t}} + {{U}}( {{{{D}}}_2{{h}}} )} ]\\
&&+ {{{a}}}_2[ {\ln {{t}} + {{U}}( {{{{D}}}_2{{h}}} )} ] + {{{a}}}_1[ {\ln {{t}} + {{U}}( {{{{D}}}_1{{h}}} )} ] = 0,\\
\end{eqnarray*}

where ${{t}} = {{T}}/{{{T}}}_{{ c}}$ is the reduced temperature, ${{h}} = \hbar {{{\mu }}}_0{{{H}}}_{{{c}}2}/2 {\phi}_0{{{k}}}_{{B}}{{T}}$ is the dimensionless magnetic field with ${\phi}_0$ the magnetic flux quantum, and ${{{D}}}_{{n}}$ is the charge carriers diffusion coefficient. The coefficients *a*_0_-*a*_2_ are defined as ${{{a}}}_0 = 2{{\omega }}/{{{\lambda }}}_0$, ${{{a}}}_1 = 1 + {{{\lambda }}}_ - /{{{\lambda }}}_0$, ${{{a}}}_2 = 1 - {{{\lambda }}}_ - /{{{\lambda }}}_0$ and ${{{\lambda }}}_ - = {{{\lambda }}}_{11} - {{{\lambda }}}_{22}$, ${{{\lambda }}}_0 = \sqrt {{{\lambda }}_ - ^2 + 4{{{\lambda }}}_{12}{{{\lambda }}}_{21}} $, ${{\omega }} = {{{\lambda }}}_{11}{{{\lambda }}}_{22} - {{{\lambda }}}_{12}{{{\lambda }}}_{21}$ where **${{\lambda }}$** is the superconducting coupling constants matrix. ${{U}}( {{x}} ) = {{\psi }}( {1/2 + {{x}}} ) - {{\psi }}( {1/2} )$, where ${{\psi }}( {{x}} )$ is the di-gamma function. The dimensionless parameters ${{{a}}}_0$, ${{{a}}}_1$, and ${{{a}}}_2$ describe the effective intraband and interband pairing interactions, while the scaled diffusivities ${{{D}}}_1$ and ${{{D}}}_2$ reflect the distinct carrier mobilities and scattering rates for the two active bands. The pronounced disparity in the extracted diffusivities (${{{D}}}_1 \gg {{{D}}}_2$) signifies a strong interband asymmetry, which effectively suppresses the pair-breaking effect in the stronger band and leads to the robust upper critical field observed in the R-I phase. All extracted fitting parameters at selected pressures are summarized in Table S4.

### Resistance fitting

To probe the lattice dynamics, we analyzed the normal-state resistance by the following equation [18,26]:


(4) \begin{eqnarray*}
{{R}}\left( {\boldsymbol{T}} \right) &=& {{{R}}}_0 + {{{R}}}_{{\rm D}}{\left( {\frac{{\boldsymbol{T}}}{{{{{\Theta }}}_{{\rm D}}}}} \right)}^5\\
&&\times \mathop \int \nolimits_0^{{{{\Theta }}}_{{\rm D}}/{{T}}} \frac{{{{{z}}}^5{{dz}}}}{{\left( {{{{e}}}^{{z}} - 1} \right)\left( {1 - {{{e}}}^{ - {{z}}}} \right)}}\\
&&+\, {{{R}}}_{{\rm E}}{\left[ {\frac{{{T}}}{{{{{\Theta }}}_{{\rm E}}}}{{\sinh }}^2\left( {\frac{{{{{\Theta }}}_{{\rm E}}}}{{2{{T}}}}} \right)} \right]}^{ - 1},
\end{eqnarray*}

where the first term ${{{R}}}_0$ is the residual resistance, and the second and third terms are the Bloch-Grüneisen and Einstein expressions, which represent the electron scattering by acoustic and optical phonons, respectively. ${{{R}}}_{{\rm D}}$ and ${{{R}}}_{{\rm E}}$ are resistance constant and ${{{\Theta }}}_{{\rm D}}$ and ${{{\Theta }}}_{{\rm E}}$ are Debye and Einstein temperatures, respectively. The fitting results of ambient sample and CAC s1 are shown in Fig. S2a and c.

### DFT calculations

The system was theoretically calculated by employing the first principles method as implemented in QUANTUM ESPRESSO (QE) package [44], and the equilibrium structure, phonon dispersions, EPC, and superconductivity of the compound were obtained as a function of the external hydrostatic pressure from 0 to 45 GPa. The plane-wave kinetic-energy cutoff and the energy cutoff for charge density were set as 100 and 400 Ry, respectively. The Brillouin zone (BZ) was sampled by a k-point mesh of 8 *× * 8 *× * 8, and a Methfessel-Paxton smearing width of 0.02 Ry was adopted for calculating the self-consistent electron density. The dynamic and EPC matrix elements were computed with an 8 *× * 8 *× * 8 *q* mesh. The phonon and EPC were evaluated quantitatively by employing the density functional perturbation theory (DFPT) and Eliashberg theory [45,46].

The electronic structure, including the DOS, was calculated using the Vienna Ab initio Simulation Package (VASP) [47]. The exchange-correlation interaction was described using the Perdew-Burke-Ernzerhof functional within the generalized gradient approximation [48], and the electron-ion interaction was treated using the projector-augmented-wave method [49]. The plane-wave energy cutoff was set to 520 eV. The Brillouin zone was sampled using automatically generated k-point meshes with a target grid density of 400 k-points per Å^−3^ of reciprocal-cell volume. COHP and integrated COHP (ICOHP) analyses were performed using the LOBSTER code [50] to evaluate the pressure evolution of the covalent bonding strength of the Re-O bonds. The wave functions used for the LOBSTER calculations were obtained from the corresponding VASP calculations. To ensure the convergence of the wave functions, the electronic self-consistent-field cycles were converged until the total-energy difference between successive steps was less than 10^−7^ eV, and no symmetry operations were imposed in these calculations.

### Magnetic susceptibility measurement

Magnetic susceptibility measurements were performed in an Magnetic Property Measurement System from Quantum Design. For the initial measurements (sample s4), a miniature non-magnetic BeCu-type DAC with a 400 μm culet and a BeCu gasket pre-compressed to 5 GPa were used, with the initial sample chamber thickness being ∼ 60 μm. To quantitatively evaluate the bulk nature of pressure-induced superconductivity in the R-I phase of ReO_3_, we estimated the superconducting volume fraction (*f*) by using the ZFC magnetic susceptibility data. For sample s4 at 23 GPa, the ZFC diamagnetic signal was measured to be *m* = −1.5 × 10^−6^ emu under an applied magnetic field of *H* = 10 Oe. The diameter of the sample was *D* ≈ 175 μm. Under 23 GPa, the sample thickness was corrected to 46 μm based on the equation of state. This yields an estimated geometric volume of *V* ≈ 1.11 × 10^−6^ cm^3^. Because the sample is approximately a flat disk (*d*/*D* ≈ 0.26) positioned perpendicular to the applied magnetic field, the demagnetization factor *N* is ∼ 0.69. The intrinsic superconducting volume fraction *f* in cgs units is derived as:


(5) \begin{eqnarray*}
{{f}} = \frac{{\frac{{4{{\pi m}}}}{{{{HV}}}}}}{{ - 1 + {{N}}\times \frac{{4{{\pi m}}}}{{{{HV}}}}}}.
\end{eqnarray*}

The superconducting volume fraction is deduced to be ∼ 78%.

To further strictly verify the bulk superconductivity at the maximum *T*_c_ dome while minimizing non-hydrostatic effects, we performed a second set of measurements (sample s5) using a DAC equipped with a 300 μm culet and a rhenium (Re) gasket. For this sample, solid KBr was used as the pressure transmit medium. Due to the smaller culet size and the inclusion of the pressure medium, the sample dimensions were strictly constrained, and a larger excitation magnetic field of *H* = 50 Oe was applied to ensure a sufficient signal-to-noise ratio.

For sample s5 at 25 GPa, the measured ZFC diamagnetic signal was *m* = −1.2 × 10^−6^ emu. The sample diameter was *D* ≈ 75 μm, and the thickness was corrected to *d* ≈ 27 μm, yielding a cylindrical volume of *V* ≈ 1.19 × 10^−7^ cm^3^. With an aspect ratio of *d*/*D* ≈ 0.36, the demagnetization factor *N* is calculated to be ∼ 0.62. Applying the identical demagnetization correction formula, the intrinsic superconducting volume fraction *f* for s5 at 25 GPa reaches an astonishing ∼ 98% (107% for 30 GPa). This robust macroscopic diamagnetic response provides direct evidence of bulk superconductivity in the R-I phase.

## Supplementary Material

nwag425_Supplemental_File
