# Supplementary material for: Discovery of record-breaking high-Tc superconductivity among 5d transition-metal-oxides up to 17.8 K in ReO3
Source: Natl Sci Rev. 2026 Jul 10;13(16):nwag425. doi: 10.1093/nsr/nwag425 (PMC13499235; doi:10.1093/nsr/nwag425)
Supplement: Supplementary file 1 [file nwag425_supplemental_file.docx]

Supplemental Information

**Discovery of record-breaking high-*T*_c_ superconductivity among 5*d* transition-metal-oxides up to 17.8 K** **in ReO_3_**

Pengfei Shan,^1,2,†^ Tenglong Lu,^1,2,†^ Ziyi Liu,^1,2, †^ Yuanyuan Jiao,^1,3^ Jiajia Feng,^4^ Pengtao Yang,^1,2^ Liang Ma,^1,5^ Yoshiya Uwatoko,^6,7^ Xiaoli Dong,^1,2^ Bosen Wang,^1,2^ Bin Chen,^4^ Miao Liu,^1,2,^* Jianping Sun,^1,2,^* and Jinguang Cheng^1,2,^*

^1^*Beijing National Laboratory for Condensed Matter Physics and Institute of Physics, Chinese Academy of Sciences, Beijing 100190, China*

^2^*School of Physical Sciences, University of Chinese Academy of Sciences, Beijing 100190, China*

^3^*Faculty of Science, Wuhan University of Science and Technology, Wuhan 430065, China*

^4^*Center for High Pressure Science and Technology Advanced Research, Beijing 100193, China*

^5^*Key Laboratory of Materials Physics, Ministry of Education, School of Physics and Microelectronics, Zhengzhou University, Zhengzhou 450052, China*

^6^*Department of Advanced Materials Science, Graduate School of Frontier Sciences, University of Tokyo, Chiba 277-8581, Japan*

*^7^Department of Natural Sciences, Faculty of Science and Engineering, Tokyo City University, Tokyo 158-8557, Japan*

†: Equally contributed to this work.

*Corresponding authors. E-mails: [jpsun@iphy.ac.cn](mailto:jpsun@iphy.ac.cn); [mliu@iphy.ac.cn](mailto:mliu@iphy.ac.cn); [jgcheng@iphy.ac.cn](mailto:jgcheng@iphy.ac.cn)

**Supplementary Tables and Figures**

Table. S1 Pressure induced structure transitions of ReO_3_ reported in literature.

| Year | | 1989 | 2000 | 2002 | 2004 | 2007 | 2015 |
| --- | --- | --- | --- | --- | --- | --- | --- |
| References | | Ref [1] | Ref [2] | Ref [3] | Ref [4] | Ref [5] | Ref [6] |
| Methods | | XRD | ED-XRD | AD-XRD | ED-XRD  Neutron | ED-XRD | Raman |
| Sample | | Poly-c | Poly-c | Poly-c | Poly-c | Nano-c | Single-c |
| PTM | | / | M-E | M-E | FC-75 | M-E | M-E-W |
| Critical Pressure (GPa) | C-II | 3 | 0.6 | C-II&R-I mixture (compression: 8-18，decompression:3-8) | ~3 | / | 0.6 |
|  | M | / | 3.1 |  | / | 0.3 | 3 |
|  | R-I | 12.5 | 12.8 |  | 13.2 | 6.7 | 12.5 |
|  | R-II | / | 39.1 | 38 | / | 20.3 | / |

Note: The Raman change cannot be determined as the phase transition of the corresponding space group, but the Raman peak changes significantly under the corresponding pressure; Poly-c: Polycrystal, Nano-c: Nano crystal, Single-c: single crystal; ED-XRD: energy-resolved X-ray diffraction; AD-XRD: angle-resolved X-ray diffraction; M-E: 4:1 methyl-ethanol mixture; FC-75: a mixture of perfluorinated hydrocarbons; M-E-W: 16:3:1 methyl-ethanol-water mixture.

Table S2. Calculated structural parameters of R-I phase (C-I phase at AP) in ReO_3_.

| *P* (GPa) | | Lattice  Parameters | Atomic coordinates  (fractional) | | | | |
| --- | --- | --- | --- | --- | --- | --- | --- |
|  |  |  | Atoms | *x* | *y* | *z* | Site |
| 0 | *a* = 3.76940 Å | | Re | 0.00000 | 0.00000 | 0.00000 | 1a |
|  |  |  | O | 0.50000 | 0.00000 | 0.00000 | 3d |
| 10 | *a* = 5.09750 Å  *c* = 12.92180 Å | | Re | 0.00000 | 0.00000 | 0.00000 | 6b |
|  |  |  | O | 0.087060 | 0.333333 | 0.583333 | 18e |
| 15 | *a* = 4.91160 Å  *c* = 12.93430 Å | | Re | 0.00000 | 0.00000 | 0.00000 | 6b |
|  |  |  | O | 0.053440 | 0.333333 | 0.583333 | 18e |
| 20 | *a* = 4.78290 Å  *c* = 12.94670 Å | | Re | 0.00000 | 0.00000 | 0.00000 | 6b |
|  |  |  | O | 0.034120 | 0.333333 | 0.583333 | 18e |
| 25 | *a* = 4.6810 Å  *c* = 12.9408 Å | | Re | 0.00000 | 0.00000 | 0.00000 | 6b |
|  |  |  | O | 0.01926 | 0.333333 | 0.583333 | 18e |
| 30 | *a* = 4.6119 Å  *c* = 12.9099 Å | | Re | 0.00000 | 0.00000 | 0.00000 | 6b |
|  |  |  | O | 0.00964 | 0.333333 | 0.583333 | 18e |
| 34 | *a* = 4.5701 Å  *c* = 12.8744 Å | | Re | 0.00000 | 0.00000 | 0.00000 | 6b |
|  |  |  | O | 0.00393 | 0.333333 | 0.583333 | 18e |
| 40 | *a* = 4.5204 Å  *c* = 12.8156 Å | | Re | 0.00000 | 0.00000 | 0.00000 | 6b |
|  |  |  | O | -0.00262 | 0.333333 | 0.583333 | 18e |
| 45 | *a* = 4.4871 Å  *c* = 12.7618 Å | | Re | 0.00000 | 0.00000 | 0.00000 | 6b |
|  |  |  | O | -0.00691 | 0.333333 | 0.583333 | 18e |

Table S3. List of samples studied in the present work for electrical transport and magnetic measurements.

| Sample | Cell Type | PTM | Sample Chamber | Pressure range / GPa | Electrical  measurements | |
| --- | --- | --- | --- | --- | --- | --- |
| s1 | CAC | Glycerol | Standard four-electrode | 2 - 13 | RT |  |
| s2 | DAC | KBr |  | 1.7 - 65.1 | RT: Fig. 2a-c; MRT: Fig. 2f, Fig. S |  |
| s3 | DAC | Daphine 7373 |  | 0.5 - 44.5 | RT: Fig. S5a-c, Fig. 2e |  |
| s4 | DAC | n/a |  | 9, 18, 23 | MT: Fig. S6a |  |
| s5 | DAC | KBr |  | 19, 25, 30 | MT: Fig. S6b, c |  |

Table S4. Fitting parameters to *H*_c2_ (T) by using the WHH model and the *T*_c_ criteria of 50% normal-state resistivity at selected pressures.

| *P* / GPa | *a*_0_ | *a*_1_ | *a*_2_ | ${D_{1}\hbar}/{2\varphi_{0}k_{B}}$ | ${D_{2}\hbar}/{2\varphi_{0}k_{B}}$ | *H*_c2_ (T) |
| --- | --- | --- | --- | --- | --- | --- |
| 16.7 | 1.47(11) | 0.65(2) | 1.35(2) | 1.30(8) | 0.235(2) | 4.6(1) |
| 17.4 | 2.19(8) | 0.80(1) | 1.20(1) | 1.05(5) | 0.206(1) | 5.8(1) |
| 18.7 | 1.77(15) | 0.66(3) | 1.34(3) | 1.10(9) | 0.191(1) | 6.8(1) |
| 20.5 | 0.34(3) | 0.31(1) | 1.69(1) | 1.91(7) | 0.152(1) | 9.5(1) |
| 24.0 | 0.34(5) | 0.32(1) | 1.68(1) | 1.53(8) | 0.129(1) | 12.1(1) |
| 27.6 | 0.32(3) | 0.44(1) | 1.56(1) | 0.90(2) | 0.103(1) | 14.6(1) |

Figure. S1. High-pressure SXRD patterns of ReO_3_ at (a) 0.2-29.7 GPa and (b) 32.6-59.6 GPa. The new peaks marked by asterisks in (b) may correspond to the unidentified R-II phase.

Figure. S2. (a) Temperature-dependent resistance at ambient pressure. (b) Hall resistance curve in 20 K at ambient pressure. (c)The resistivity measured under hydrostatic pressures by using CAC, which was fitted by equation (3). The inset shows the emergence of superconductivity at low temperatures.

Figure. S3 Temperature dependence of resistance of ReO_3_ (DAC s3 along <110> axis) measured at various pressures (a) from 5.5 to 14.0 GPa, (b) from 17.1 to 31.9 GPa, and (c) from 35.0 GPa to 44.5 GPa.

Figure. S4. Temperature dependence of resistance under different magnetic fields at (a) 14.1 GPa, (b) 16.7 GPa, (c) 17.1 GPa, (d) 18.7 GPa, (e) 20.5 GPa, (f) 24.0 GPa, (g) 27.6 GPa, (h) 32.7 GPa, and (i) 40.7 GPa.

Figure. S5. Temperature dependencies of *μ_0_H*_c2_ (*T*) fitted by the WHH two-band model at selected pressures.

Figure. S6. Macroscopic magnetic evidence for bulk superconductivity in the high-pressure R-I phase of ReO_3_. (a) Temperature dependence of zero-field-cooled (ZFC) magnetization for sample s4 at selected pressures up to 23 GPa. (b) Systematic ZFC and field-cooled (FC) magnetization curves for sample s5 across the superconducting dome. (c) Field-dependent magnetization curves for sample s5 measured at the pressure of 25 GPa under varying applied magnetic fields (*H* = 10, 50, and 100 Oe)*.*

Figure. S7. Evolution of the energy-resolved COHP curves for Re-O interactions under pressure. The bonding contributions are shaded in blue, while the antibonding contributions are shaded in pink. The *E*_F_ is set to zero. As the pressure increases from 0 to 40 GPa, the significant broadening of the energy bandwidths indicates a pressure-driven enhancement in the spatial overlap and delocalization between the Re 5*d* and O 2*p* orbitals, pointing towards a highly hybridized covalent network in the R-I phase.

Figure. S8. Pressure dependence of the integrated COHP (-ICOHP) for Re-O bonds. The -ICOHP values are integrated up to the Fermi level. The monotonic increase from ambient pressure up to 40 GPa quantitatively demonstrates the continuous strengthening of the Re-O covalent bonds under external compression. This structural and electronic optimization provides an indispensable synergistic framework for the emergence of high-*T*_c_ superconductivity.

**References**

[1] Dyuzheva TI, Bendeliani NA, Glushko AN *et al.* Phase diagram of ReO_3_ up to 10 GPa. *Phys Scr* 1989; **39**: 341.

[2] Jorgensen J-E, Olsen SJ, Gerward L. Phase transitions in ReO_3_ studied by high-pressure X-ray diffraction. *J Appl Crystallogr* 2000; **33**: 279.

[3] Suzuki E, Kobayashi Y, Endo S *et al.* Structural phase transition in ReO_3_ under high pressure. *J Phys Condens Matter* 2002; **14**: 10589.

[4] Jorgensen J-E, Marshall WG, Smith RI *et al.* High-pressure neutron powder diffraction study of the *Im*-3 phase of ReO_3_. *J Appl Crystallogr* 2004; **37**: 857.

[5] Biswas K, Muthu DVS, Sood AK *et al.* Pressure-induced phase transitions in nanocrystalline ReO_3_. *J Phys Condens Matter* 2007; **19**: 436214.

[6] Muthu DVS, Teredesai P, Saha S *et al.* Pressure-induced structural phase transitions and phonon anomalies in ReO_3_: Raman and first-principles study. *Phys Rev B* 2015; **91**: 224308.
